# Oral health problems and risk of incident disability in two studies of older adults in the United Kingdom and the United States

**DOI:** 10.1111/jgs.17792

**Published:** 2022-04-19

**Authors:** Eftychia Kotronia, Heather Brown, Olia Papacosta, Lucy T. Lennon, Robert J. Weyant, Peter H. Whincup, Sasiwarang Goya Wannamethee, Sheena E. Ramsay

**Affiliations:** ^1^ Population Health Sciences Institute Newcastle University Newcastle Upon Tyne UK; ^2^ Department of Primary Care & Population Health Institute of Epidemiology and Health Care, University College London London UK; ^3^ Department of Dental Public Health, School of Dental Medicine University of Pittsburgh Pittsburgh Pennsylvania USA; ^4^ Population Health Research Institute St George's University of London London UK

**Keywords:** dental problems, disability, older adults, physical independence, self‐reported

## Abstract

**Background:**

Preventing oral health problems can be crucial for maintaining physical independence in older adults. We aimed to examine the associations of a range of oral health problems with incidence of disability in older adults.

**Methods:**

We used prospective data from the British Regional Health Study (BRHS) (*N* = 2147, 71–92 years), and the Health, Aging and Body Composition (HABC) study (USA) (*N* = 3075, 71–80 years). Oral health measures included tooth loss, periodontal disease, self‐rated oral health, and self‐reported dry mouth. Participants were followed for onset of disability over a follow‐up period of 3 years. Onset of disability was assessed through new cases of mobility limitations, activities of daily living (ADL), and instrumental activities of daily living (IADL). Logistic regression was performed to calculate the odds of incident disability.

**Results:**

In the BRHS, tooth loss was associated with greater odds of mobility limitations and ADL difficulties. Periodontal disease was associated with greater incidence of mobility limitations. Self‐report of ≥3 dry mouth symptoms was associated with increased odds of incident mobility limitations and ADL difficulties (OR = 2.08, 95% CI 1.27–3.42; OR = 1.73, 95% CI 1.03–2.90). Fair/poor self‐rated oral health was associated with greater incidence of IADL difficulties. In the HABC study, complete tooth loss was associated with greater incidence of mobility limitations (OR = 1.86, 95% CI 1.13–3.06), and fair/poor self‐rated oral health was associated with increased odds of incident ADL difficulties (OR = 1.42, 95% CI 1.04–1.94).

**Conclusions:**

Oral health problems in older adults, particularly tooth loss, self‐reported dry mouth and self‐rated oral health were associated with greater incidence of disability. Poor oral health plays a potentially important role in the development of disability in older populations, which in turn is an essential part of quality of life and healthy aging.

**Key points**

- Tooth loss was associated with greater incidence of different types of disability in older adults.
- Self‐reported oral health problems were associated with increased incidence of disability in older adults.
- Poor oral health could potentially be an important aspect of maintaining physical independence in older age.

**Why does this paper matter?**

Poor oral health is a potentially important factor contributing to disability in older populations, and therefore limiting physical independence, a factor which is key to quality of life.

## INTRODUCTION

Worldwide, around 17% of the total population will be ≥65 years by 2050.^1^ Hence, older individuals will be more likely to experience disability, which constitutes a significant impediment to physical independence.^2^, ^3^ Disability is associated with chronic diseases and poor quality of life in older community‐dwelling adults.^4^, ^5^, ^6^, ^7^ Poor oral health is very prevalent in older people in the United Kingdom and the United States^8^, ^9^ and is an integral part of the health of older populations.^10^ It can have significant adverse effects on eating, resulting in a poor diet and quality of life.^11^, ^12^ Poor oral health can influence chronic diseases, including cardiovascular disease, diabetes, cognitive function, and mortality.^13^, ^14^, ^15^

In older populations, bidirectional associations exist between poor oral health and disability, with both health outcomes influencing one another. One potential direction is that poor oral health, specifically tooth loss, can influence the development of disability, including activities of daily living (ADL), instrumental activities of daily living (IADL), and mobility limitations, in older age.^16^, ^17^, ^18^, ^19^, ^20^, ^21^ Nevertheless, limited evidence exists for the associations between oral health and incidence of mobility limitations, defined by difficulty walking and going up stairs, which is a key feature of functional independence.^22^ Little is also known about the association of periodontal disease and self‐reported oral health measures with incident disability in older adults. Periodontal disease is a chronic oral disease, which is associated with oral inflammation and poor diet quality, which can influence muscle strength and disability.^23^, ^24^, ^25^ Additionally, self‐reported oral health measures, including poor self‐rated oral health, and self‐reported dry mouth are important indicators of the burden of periodontal disease and tooth loss in older adults^26^, ^27^, ^28^ and may impact the development of disability. Therefore, we aimed to examine the prospective associations of several oral health measures, both clinical and self‐reported, with the incidence of mobility limitations, ADL and IADL difficulties in two population‐based studies of older community‐dwelling adults living in the United Kingdom and the United States. Including two population‐based studies allowed us to investigate these associations in two comparable and complementary studies of older populations. Additionally, we aimed to examine whether similar associations exist in the two studies, which consist of different populations of older people; the BRHS includes white British men, whereas the HABC study comprises white and African American men and women in the United States. Presence of similar associations in both studies would strengthen evidence of an association.

## METHODS

### The British Regional Heart Study

This is an ongoing cohort study, which started in 1978–1980 and comprised 7735 white British men aged 40–59 years. Individuals were recruited from 24 towns across the United Kingdom in 1978–1980 and have been followed‐up since.^29^ For this study, data from the 30‐year follow‐up (2010–2012) and examination in 2014 were used. The 30‐year follow‐up of the cohort was undertaken in 2010–2012 and 3137 surviving participants were invited to participate. In total, 2147 participants aged 71–92 years completed a postal questionnaire (68% response rate), and 1722 attended a physical and oral health examination (55% response rate) and had blood samples taken.^29^ Oral examination was conducted by a trained nurse. Participants were followed‐up for disability from 2010–2012 to 2014. To calculate new cases of disability, those reporting disability at baseline (2010–2012) (*n* = 564) and those who did not answer any disability‐related questions were excluded (*n* = 44), resulting in a sample size of 1153. Ethical approval was provided by the National Research Ethics Service Committee, London. Written informed consent was obtained from individuals for their participation in the study, according to the Declaration of Helsinki.

### The Health, Aging and Body Composition study

The HABC study is a prospective population‐based study investigating deterioration in physical function of older individuals and how changes in body composition influence health in older age. The cohort was initially examined in 1997–1998, where 3075 white and African American males and females aged 70–79 years were recruited. Random selection of white participants was performed through Medicare, whereas African American were selected through neighborhoods with a ZIP code around Memphis and Pittsburgh.^30^ Individuals who were not able to walk 0.25 miles or climb 10 steps were excluded from the study at baseline. For this study, data from Year 2 (1998–1999) and Year 5 (2001–2002) were used. In Year 2, 2998 males and females aged 71–80 years (response rate = 97.5%) answered questionnaires, attended oral health (*n* = 1975) and physical assessments, and provided blood samples. Oral examination was conducted by a dental hygienist or periodontist. Follow‐up for disability was carried out from 1998–1999 to 2001–2002. To calculate new cases of disability those who reported disability in 1998–1999 were excluded (*n* = 882), resulting in a sample size of 1913. All participants provided written informed consent. Ethical approval was provided by several institutional review boards.^30^

## ORAL HEALTH

In both studies, clinical measures of oral health were assessed through an oral examination, whereas self‐reported measures were assessed through questionnaires. Clinical measures of oral health comprised count of remaining natural teeth (tooth loss) and periodontal disease (loss of attachment and pocket depth in participants with teeth). Tooth loss categories reflected the number of natural teeth remaining in the mouth. In the HABC study, to assess periodontal disease, a full mouth assessment was performed by a dental hygienist or a periodontist with 90% agreement between examiners, which was ascertained before the start of the dental examination,^31^ whereas in the BRHS periodontal, measurements were carried out in six index teeth by trained research nurses with a range of agreement between the examiner and the trainer from 89% to 95%, and a median κ index equal to 0.79.^32^ Further details about the assessment of loss of attachment and pocket depth can be found elsewhere.^31^, ^32^ Self‐reported measures of oral health included self‐rated oral health, dry mouth, difficulty eating, sensitivity to hot/cold/sweets (BRHS), and limitation of food due to gum problems (HABC study). In the HABC study, one question was used to assess dry mouth (dry mouth when eating); in the BRHS, the Xerostomia Inventory Scale was used to measure dry mouth symptoms.^33^ In both studies, a composite measure of the presence of more than one (cumulative) oral health problems was created, to assess potential associations with incidence of disability later in life.

## DISABILITY

Disability comprised mobility limitations, ADL, and IADL. In the BRHS, mobility limitations were defined as difficulty going up or down stairs or walking 400 yards. Individuals facing difficulty performing either one or both activities were classified as having mobility limitations. To measure ADL difficulties, participants were asked whether they faced difficulties with any of the following activities: getting in and out of a chair, dressing and undressing yourself, bathing or showering, feeding yourself, including cutting food, or getting to and using the toilet. Participants with difficulty in performing at least one activity were classified as having ADL difficulties.^34^ Similarly, for IADL difficulties, individuals experiencing difficulty with any of the following activities: shopping for personal items, preparing your own meals, using telephone by yourself, managing money, or using public transport, were classified as having IADL difficulties.^34^ In the HABC study, participants experienced mobility limitations when they either faced difficulty walking 1 quarter of a mile or climbing 1 flight of stairs or both. For ADL, participants experiencing difficulty with at least one of the following activities: dressing, getting in and out of bed, and/or bathing on your own, were classified as having ADL difficulties.^34^ Information on IADL was not collected in the HABC study. Details about the assessment of disability in both studies can be found in the Text S2.

## COVARIATES

Covariates were assessed at the 30‐year re‐examination in 2010–2012 in the BRHS and at Year 2 in 1998–1999 in the HABC Study (see Table S1). In both studies, information on socioeconomic status, smoking, alcohol, physical activity, history of cardiovascular disease (CVD), and history of diabetes was provided through questionnaires. In the HABC study, socioeconomic position was defined according to years of education, whereas in the BRHS, socioeconomic position was assessed by social class (longest held occupation when entering the study).^32^ In the HABC study, physical activity was based on the sum of calories spent weekly for various activities. In the BRHS, physical activity was classified as inactive, occasional, light moderate, moderate, moderately vigorous. In both studies, body weight and height were measured and used to calculate body mass index (BMI). Moreover, medications causing xerostomia (dry mouth) and depression (see Supplemental Text S3) were identified.

## STATISTICAL ANALYSIS

In both studies, to examine the associations of oral health problems with incidence of disability, logistic regression was performed separately for each study. Effect estimates are presented as odds ratios (OR) and 95% confidence intervals (CIs). In the BRHS, regression models were adjusted for age, social class, smoking, alcohol, physical activity, history of CVD and diabetes, and BMI. In the HABC study, models were adjusted for age, gender, race, education, smoking, alcohol, physical activity, history of CVD and diabetes, and BMI.

## RESULTS

Baseline characteristics and prevalence of oral health problems in both studies are presented in Table 1. In the BRHS, median age was 77.7 years, 52% were in nonmanual occupation class, 36% were never smokers and 4% were current smokers, 36% consumed alcohol almost every day, 20% were inactive, 24% had history of CVD, 16% had history of diabetes, and 20% obese. In the HABC study, median age was 74.7 years, 52% were female, 42% were African American, 42% had completed postsecondary education, 10% were current smokers and 46% were ex‐smokers, 7% consumed alcohol almost daily, 64.5 kcal/kg/week were spent on exercise, 4% had history of CVD, 5% had history of diabetes, and 24% were obese.

**TABLE 1:** Population characteristics at baseline in the British Regional Health Study (BRHS) and Health, Aging and Body Composition (HABC) study populations

BRHS (*n* = 2147)		HABC Study (*n* = 2998)	
**Age (years),** Health, Aging and Body Composition median (IQR)	77.7 (74.8–82.1)	**Age (years),** median (IQR)	74 (72–77)
		**Gender,** n (%)	
		Male	1491 (48)
		Female	1584 (52)
		**Race,** n (%)	
		White	1794 (58)
		African American	1281 (42)
**Social class,** n (%)		**Education,** n (%)	
Nonmanual	1081 (52)	Less than high school	775 (25)
Manual	1003 (48)	High school graduate	1000 (33)
		Postsecondary	1292 (42)
**Smoking,** *n* (%)		**Smoking,** *n* (%)	
Never	768 (36)	Never	1348 (44)
Current smoker	91 (4)	Current smoker	318 (10)
Ex‐smoker	1275 (60)	Ex‐smoker	1401 (46)
**Alcohol,** *n* (%)		**Alcohol,** *n* (%)	
None	292 (36)	No consumption past year	1546 (50)
Occasionally	307 (15)	1–7 times per week	655 (21)
Daily	757 (14)	More than 1 per day	227 (7)
**Physical activity,** *n* (%)		**Physical activity (kcal/kg/week),** median (IQR)	64.5 (38.3–106.5)
Inactive	405 (20)		
Occasional	475 (24)		
Moderate	278 (14)		
**History of cardiovascular disease,** *n* (%)	500 (24)	**History of cardiovascular disease,** *n* (%)	106 (4)
**History of diabetes,** *n* (%)	321 (15)	**History of diabetes,** *n* (%)	142 (5)
**Body Mass Index,** *n* (%)		**Body Mass Index,** *n* (%)	
Normal	486 (29)	Normal	963 (34)
Overweight	875 (51)	Overweight	1192 (42)
Obese	343 (20)	Obese	673 (24)
**Depression,** *n* (%)		**Depression,** *n* (%)	
Yes	194 (10)	Yes	89 (3)
No	1727 (90)	No	2840 (97)
**Oral health measures**, *n* (%)		**Oral health measures**, *n* (%)	
Complete tooth loss (0 teeth)	338 (20)	Complete tooth loss (0 teeth)	208 (10)
Partial tooth loss (<21 teeth)	1066 (64)	Partial tooth loss (<21 teeth)	1031 (52)
>20% sites with loss of attachment >3.5 mm	303 (24)	>20% sites with loss of attachment ≥3 mm	721 (64)
>20% sites with pocket depth >5.5 mm	365 (29)	>20% sites with pocket depth ≥3 mm	627 (55)
Fair/poor self‐rated oral health	719 (35)	Fair/poor self‐rated oral health	829 (31)
≥1 dry mouth symptoms	1272 (62)	Dry mouth when eating	107 (4)
≥2 oral health problems	766 (36)	≥2 oral health problems	617 (22)

With regards to oral health problems, in the BRHS, 20% of the participants had no natural teeth, 24% had >20% of sites with loss of attachment, 35% reported fair/poor self‐rated oral health, 62.5% had at least 1 dry mouth symptom (self‐reported), and 36% had at least 2 oral health problems. In the HABC study, 10.5% had no natural teeth, 64% had >20% of sites with loss of attachment, 30.5% reported fair/poor self‐rated oral health, 4% had dry mouth symptoms when eating (self‐reported), and 22% had at least 2 oral health problems.

## ASSOCIATIONS OF ORAL HEALTH PROBLEMS WITH INCIDENT MOBILITY LIMITATIONS

**FIGURE 1:**
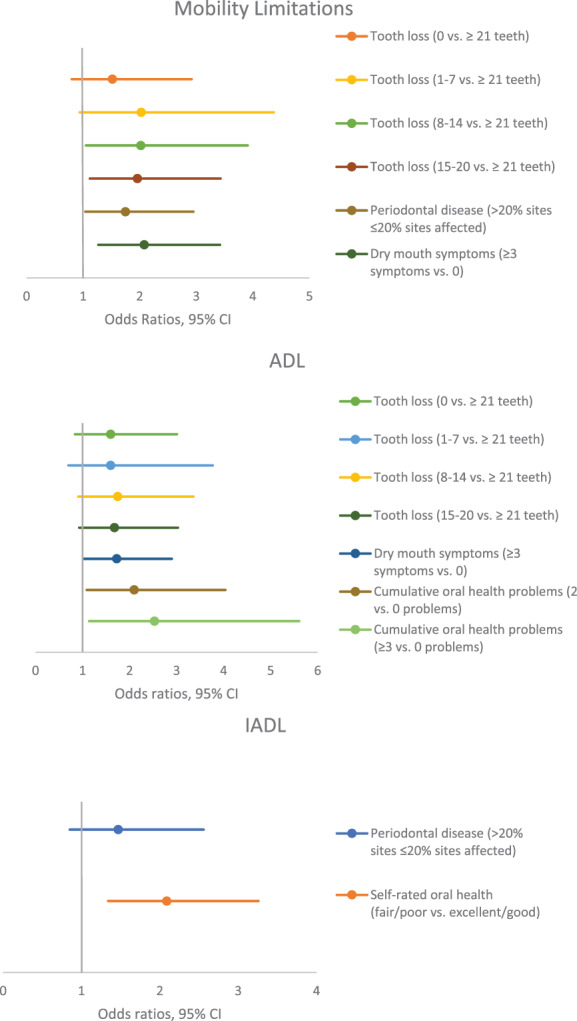
Odds ratios and 95% confidence interval (CI) for the associations between oral health measures and incident mobility limitations, activities of daily living (ADL), and instrumental activities of daily living (IADL) difficulties in older people in the BRHS. Not all study findings are shown in this figure. Please see Table 2 for a detailed presentation of associations between oral health problems and incident mobility limitations, ADL, and IADL difficulties in the BRHS

Odds ratios (OR) and 95% CI for the associations between poor oral health and incident mobility limitations in the BRHS are presented in Table 2. An overview of findings can be found in Figure 1. In the BRHS, partial tooth loss (having 8–14, 15–20 natural teeth vs. ≥21 teeth) was associated with greater incidence of mobility limitations (OR = 2.02, 95% CI 1.05–3.91; OR = 1.96, 95% CI 1.12–3.43, respectively) after adjustment for age, social class, smoking, alcohol, physical activity, history of CVD and diabetes, and BMI. Complete tooth loss was not associated with incident mobility limitations. Additionally, periodontal disease was associated with greater incidence of mobility limitations after full adjustment. Self‐report of at least 3 dry mouth symptoms was also associated with higher incidence of mobility limitations after adjusting for confounders.

**TABLE 2:** Odds ratios (OR) and 95% CI for the associations of oral health problems with incident mobility limitations, ADL and IADL difficulties in older British men in the British Regional Health Study (BRHS)

	Incident mobility limitations (*n* = 175; 15%)		
	N (%)	Age adjusted	Fully adjusted^a^
		OR (95% CI)	OR (95% CI)
**Tooth loss** (N of teeth)			
≥21	34 (9%)	1.00	1.00
15–20	35 (17%)	2.02 (1.21, 3.36)	1.96 (1.12, 3.43)
8–14	33 (22%)	2.75 (1.62, 4.66)	2.02 (1.05, 3.91)
1–7	13 (21%)	2.22 (1.08, 4.56)	2.03 (0.94, 4.37)
0	30 (18%)	2.02 (1.18, 3.47)	1.52 (0.80, 2.92)
**Periodontal disease (% of sites with loss of attachment >5.5 mm)** ^b^			
<20%	71 (12%)	1.00	1.00
≥20%	38 (21%)	1.82 (1.17, 2.83)	1.75 (1.04, 2.95)
**Self‐rated oral health**			
Good/Excellent	104 (14%)	1.00	1.00
Fair/Poor	67 (19%)	1.41 (1.00, 1.97)	0.97 (0.64, 1.49)
**Dry mouth symptoms**			
0	49 (11%)	1.00	1.00
1–2	62 (16%)	1.52 (0.91, 2.53)	1.36 (0.84, 2.20)
≥3	59 (22%)	2.50 (1.45, 4.30)	2.08 (1.27, 3.42)
**Cumulative oral health problems** ^c^			
0	21 (9%)	1.00	1.00
1	78 (14%)	1.54 (0.93, 2.57)	1.70 (0.99, 2.91)
2	53 (21%)	2.52 (1.46, 4.35)	1.43 (0.71, 2.87)
≥3	23 (24%)	3.01 (1.57, 5.79)	1.83 (0.70, 4.80)
**Tooth Loss** (N of teeth)		**Incident ADL** (n = 122; 11%)	
≥21	31 (7%)	1.00	1.00
15–20	29 (12%)	1.70 (0.99, 2.91)	1.68 (0.93, 3.03)
8–14	26 (16%)	2.19 (1.24, 3.84)	1.75 (0.91, 3.36)
1–7	10 (14%)	1.76 (0.81, 3.83)	1.62 (0.70, 3.77)
0	26 (14%)	1.64 (0.93, 2.89)	1.60 (0.84, 3.01)
**Periodontal disease (% of sites with loss of attachment >5.5 mm)** ^b^			
<20%	63 (10%)	1.00	1.00
≥20%	29 (15%)	1.50 (0.93, 2.43)	1.12 (0.64, 1.94)
**Self‐rated oral health**			
Good/Excellent	84 (10%)	1.00	1.00
Fair/Poor	63 (16%)	1.63 (1.14, 2.32)	1.16 (0.76, 1.80)
**Dry mouth symptoms**			
0	44 (9%)	1.00	1.00
1–2	48 (11%)	1.25 (0.81, 1.94)	1.06 (0.64, 1.76)
≥3	51 (16%)	1.90 (1.23, 2.93)	1.73 (1.03, 2.90)
**Cumulative oral health problems** ^c^			
0	20 (8%)	1.00	1.00
1	59 (10%)	1.18 (0.69, 2.01)	1.18 (0.64, 2.20)
2	49 (17%)	2.35 (1.35, 4.08)	2.10 (1.09, 4.04)
≥3	22 (20%)	2.63 (1.36, 5.08)	2.53 (1.14, 5.61)
**Tooth Loss** (N of teeth)		**Incident IADL** (n = 159; 12%)	
≥21	36 (8%)	1.00	1.00
15–20	28 (12%)	1.39 (0.82, 2.35)	1.51 (0.83, 2.74)
8–14	24 (14%)	1.60 (0.92, 2.80)	1.48 (0.77, 2.84)
1–7	8 (11%)	1.10 (0.48, 2.51)	0.88 (0.33, 2.32)
0	34 (18%)	2.02 (1.20, 3.40)	1.56 (0.84, 2.91)
**Periodontal disease (% of sites with loss of attachment >5.5 mm)** ^b^			
<20%	62 (9%)	1.00	1.00
≥20%	27 (13%)	1.35 (0.82, 2.20)	1.47 (0.85, 2.56)
**Self‐rated oral health**			
Good/Excellent	83 (10%)	1.00	1.00
Fair/Poor	72 (18%)	1.92 (1.36, 2.71)	2.09 (1.34, 3.26)
**Dry mouth symptoms**			
0	54 (11%)	1.00	1.00
1–2	57 (13%)	1.30 (0.87, 1.95)	1.21 (0.73, 2.01)
≥3	45 (14%)	1.33 (0.86, 2.04)	1.33 (0.78, 2.27)
**Cumulative oral health problems** ^c^			
0	18 (7%)	1.00	1.00
1	87 (14%)	2.00 (1.17, 3.40)	1.27 (0.68, 2.35)
2	38 (13%)	1.85 (1.02, 3.34)	1.30 (0.65, 2.60)
≥3	18 (16%)	2.40 (1.19, 4.85)	1.81 (0.78, 4.23)

Abbreviations: ADL, Activities of Daily Living; IADL, Instrumental Activities of Daily Living.

^a^ Adjusted for age, social class, smoking, alcohol, physical activity, history of CVD and diabetes, BMI.

^b^ No associations were observed between pocket depth and incidence of disability; therefore only effect estimates for loss of attachment are presented.

^c^ <21 teeth, ≥3 dry mouth symptoms, difficulty eating, sensitivity to hot/cold/sweets.

**FIGURE 2:**
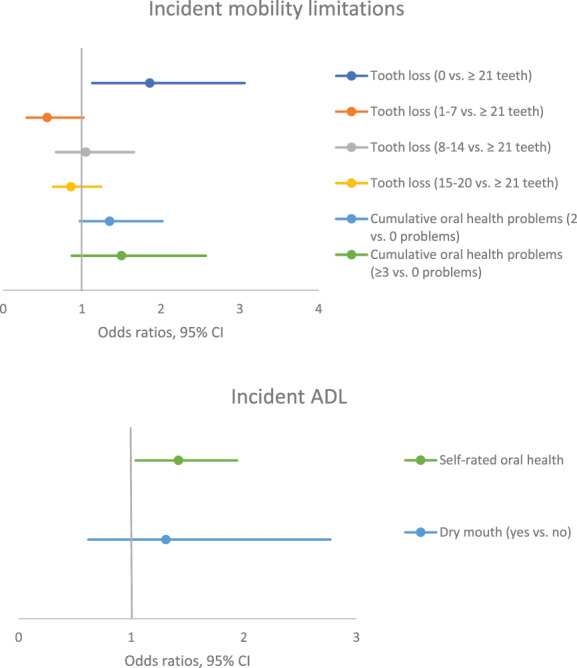
Odds ratios and 95% confidence interval (CI) for the associations between oral health measures and incident mobility limitations, and activities of daily living (ADL) difficulties in older people in the HABC study. Not all study findings are shown in this figure. Please see Table 3 for a detailed presentation of associations between oral health problems and incident mobility limitations and ADL difficulties in the HABC study

ORs and 95% CI for the associations between poor oral health and incident mobility limitations in the HABC study are presented in Table 3. An overview of findings can be found in Figure 2. In the HABC study, complete tooth loss (0 vs. having ≥21 natural teeth) was associated with greater odds of developing mobility limitations (OR = 1.86, 95% CI 1.13–3.06) after adjustment for age, gender, race, smoking, alcohol, physical activity, history of CVD and diabetes, and BMI. Fair/poor self‐rated oral health and cumulative oral health problems were associated with greater incidence of mobility limitations only in the age adjusted models. No further associations were observed in the HABC study.

**TABLE 3:** Odds ratios (OR) and 95% CI for the association of oral health problems with incident mobility limitations and ADL difficulties in older US men and women in the Health, Aging and Body Composition (HABC) study

	Incident mobility limitations (n = 380; 20%)			Incident ADL (n = 250; 13%)		
	N (%)	Age adjusted	Fully adjusted^a^	N (%)	Age adjusted	Fully adjusted^a^
		OR (95% CI)	OR (95% CI)		OR (95% CI)	OR (95% CI)
**Tooth loss** (N of teeth)						
≥21	111 (16%)	1.00	1.00	84 (12%)	1.00	1.00
15–20	42 (17%)	1.05 (0.71, 1.55)	0.86 (0.57, 1.31)	31 (12%)	1.01 (0.65, 1.58)	0.80 (0.50, 1.28)
8–14	40 (23%)	1.52 (1.01, 2.29)	1.05 (0.67, 1.66)	23 (14%)	1.15 (0.70, 1.89)	0.89 (0.52, 1.52)
1–7	17 (15%)	0.90 (0.52, 1.56)	0.56 (0.30, 1.02)	11 (9%)	0.70 (0.36, 1.36)	0.58 (0.29, 1.15)
0	38 (31%)	2.33 (1.51, 3.60)	1.86 (1.13, 3.06)	17 (13%)	1.05 (0.60, 1.84)	0.77 (0.41, 1.44)
**Periodontal disease (% of sites with loss of attachment >3 mm)** ^b^						
<20%	49 (16%)	1.00	1.00	31 (10%)	1.00	1.00
≥20%	73 (15%)	0.92 (0.62, 1.37)	0.85 (0.56, 1.31)	54 (10%)	0.99 (0.62, 1.58)	0.90 (0.55, 1.49)
**Self‐rated oral health**						
Good/Excellent	238 (18%)	1.00	1.00	146 (11%)	1.00	1.00
Fair/Poor	106 (22%)	1.34 (1.03, 1.73)	1.09 (0.83, 1.45)	81 (16%)	1.59 (1.19, 2.14)	1.42 (1.04, 1.94)
**Dry mouth**						
No	331 (19%)	1.00	1.00	218 (12%)	1.00	1.00
Yes	13 (27%)	1.63 (0.85, 3.13)	1.45 (0.73, 2.89)	9 (16%)	1.41 (0.68, 2.92)	1.31 (0.62, 2.77)
**Cumulative oral health problems** ^c^						
0	87 (15%)	1.00	1.00	66 (12%)	1.00	1.00
1	174 (19%)	1.28 (0.97, 1.70)	0.98 (0.72, 1.33)	109 (12%)	1.02 (0.74, 1.42)	0.83 (0.58, 1.17)
2	62 (25%)	1.85 (1.28, 2.68)	1.35 (0.91, 2.02)	36 (15%)	1.33 (0.86, 2.07)	1.08 (0.68, 1.71)
≥3	27 (28%)	2.12 (1.29, 3.50)	1.50 (0.87, 2.57)	21 (18%)	1.68 (0.98, 2.87)	1.27 (0.72, 2.26)

Abbreviation: ADL: Activities of Daily Living.

^a^ Adjusted for age, gender, race, education, smoking, alcohol, physical activity, history of CVD and diabetes, BMI.

^b^ No associations were observed between pocket depth and incidence of disability; therefore only effect estimates for loss of attachment are presented.

^c^ <21 teeth, dry mouth when eating, difficulty eating or chewing, limitation of food due to gum problems.

## ASSOCIATIONS OF ORAL HEALTH PROBLEMS WITH INCIDENT ADL DIFFICULTIES

ORs and 95% CI for the associations between poor oral health and incident ADL difficulties in the BRHS are presented in Table 2. An overview of findings can be found in Figure 1. In the BRHS, partial tooth loss (having <21 vs. ≥21 teeth) was associated with greater odds of developing ADL difficulties (OR = 1.68, 95% CI 1.02–2.73) after adjusting for confounders. No associations were reported between complete tooth loss and incidence of ADL difficulties. Fair/poor self‐rated oral health was associated with increased incidence of ADL difficulties in the age adjusted model but was attenuated and did not remain after adjustment. Additionally, self‐report of ≥3 dry mouth symptoms (OR = 1.73, 95% CI 1.03–2.90) and cumulative oral health problems (≥3 vs. 0 problems, OR = 2.53, 95% CI 1.14–5.61) were associated with increased odds of developing ADL difficulties after 4 years of follow‐up in the fully adjusted models.

ORs and 95% CI for the associations between poor oral health and incident ADL difficulties in the HABC study are presented in Table 3. An overview of findings can be found in Figure 2. We did not observe associations between tooth loss, periodontal disease, and incidence of ADL difficulties. Fair/poor self‐rated oral health was associated with greater incidence of ADL difficulties (OR = 1.42, 95% CI 1.04–1.94) after adjusting for confounders. No associations were observed between self‐reported dry mouth, cumulative oral health problems, and increased incidence of ADL difficulties.

## ASSOCIATIONS OF ORAL HEALTH PROBLEMS WITH INCIDENT IADL DIFFICULTIES

This analysis could only be performed in the BRHS. ORs and 95% CI for the associations between poor oral health and incident IADL difficulties in the BRHS are presented in Table 2. An overview of findings can be found in Figure 1. In the BRHS, complete and partial tooth loss were associated with greater odds of incident IADL difficulties only in the age adjusted models. We did not observe any associations between periodontal disease and development of IADL difficulties. Moreover, fair/poor self‐rated oral health was associated with higher incidence of IADL difficulties (OR = 2.09, 95% CI 1.34–3.26) in the fully adjusted model. No associations were reported between self‐reported dry mouth and incident IADL difficulties. Cumulative oral health problems were associated with greater odds of developing IADL difficulties only in the age‐adjusted model.

## DISCUSSION

Poor oral health showed limited significant associations with the incidence of disability in later life. The majority of significant associations were observed in the BRHS.

In the BRHS only, tooth loss was associated with incident mobility limitations, which can be a predictor of ADL and IADL and indicator of poor physical function.^22^, ^35^, ^36^ It is possible that chewing difficulties, through influencing eating and food choices could contribute to decreased muscle strength, which then impacts physical function and stability and can lead to onset of disability.^25^, ^37^, ^38^, ^39^ This pathway of eating difficulties was highlighted by a recent study, where the reported association between tooth loss and incident ADL disability was explained (10%) by self‐reported eating difficulties in an older population.^19^ Furthermore, only in the BRHS, tooth loss was associated with greater incidence of ADL difficulties, in accordance with previous studies.^18^, ^19^, ^40^

Additionally, only in the BRHS, periodontal disease was associated with greater incidence of mobility limitations. Periodontal disease is a chronic inflammatory disease and is associated with elevated oral and systemic inflammation.^41^, ^42^ Inflammation has been suggested as one potential mediator in this association, with oral inflammation caused by periodontal disease leading to loss of muscle mass and muscle strength, therefore contributing to decreased ability to perform everyday activities or move around.^43^ Moreover, only in the BRHS, we observed that self‐reported dry mouth was associated with greater incidence of mobility limitations and ADL difficulties. We have previously shown cross‐sectional associations between dry mouth and disability;^34^ however, there were no studies identified examining this association prospectively, and this study adds valuable information about the associations of self‐reported dry mouth and onset of disability. Dry mouth can be an indicator of tooth loss and periodontal disease and can exacerbate already existing oral health problems.^26^ Additionally, dry mouth may influence the development of disability through (a) it's association with oral inflammation similar to periodontal disease, and (b) it's influence on eating behaviors, resulting in food avoidance and poor diet quality, and therefore contribute to the development of disability.^26^, ^44^ Dry mouth can also be caused by medications prescribed for chronic diseases and thus being an indirect indicator of overall health status.^45^ However, in our analysis, we did not observe any change in associations even when accounting for medications causing xerostomia (dry mouth).

Fair/poor self‐rated oral health was associated with greater incidence of ADL (HABC study) and IADL difficulties (BRHS) in older people, as suggested by a previous study.^40^ Poor oral health including tooth loss and periodontal disease can affect self‐rating of oral health and can indicate presence of untreated dental problems.^46^ The reported association shows that self‐rated oral health may reflect future deterioration of physical function, onset of disability and potentially worsening general health.

Finally, only in the BRHS, cumulative oral health problems were associated with higher incidence of ADL difficulties. This finding shows that impaired oral health can have a significant impact on physical independence in older age.

Depression has been associated with poor oral health (e.g., tooth loss, periodontal disease, dental caries), and antidepressant medications can cause dryness of mouth.^47^ Likewise, depression can also be a common cause of disability.^48^ When analyses were adjusted for depression, in the BRHS, associations of periodontal disease with incident mobility limitations and of self‐reported dry mouth with incident ADL were no longer significant, while associations of self‐reported dry mouth with incident mobility limitations and of self‐rated oral health with incident IADL difficulties were weakened but remained significant (see Table S2). It is possible that self‐report of oral health can be affected by depression in older people. Nevertheless, in the HABC study, associations did not change (see Table S3), which can be explained by the low prevalence of depression.

### Strengths and limitations

One of the main strengths of this study is that prospective associations of a comprehensive set of oral health problems with the onset of disability were examined in two large population‐based cohort studies of older community‐dwelling adults living in the United Kingdom and the United States. Additionally, disability was assessed comprehensively through three different measures, and similar covariates were measured in the two studies. Both studies included comparable oral health data, both clinical and self‐reported. However, this study has some limitations. Findings from both cohorts may not be generalizable to the whole population of older people in the United Kingdom and the United States because BRHS consisted of white British men living across the United Kingdom and the HABC study included white and African American men and women living in Pittsburgh and Memphis, USA. Furthermore, some oral health problems (periodontal disease, dry mouth) were assessed differently in the two studies, which may explain the different findings. Moreover, differences existed in the assessment of ADL between the two studies, which could explain differences in associations between poor oral health and incident ADL. Additionally, participants of the two studies differed according to lifestyle factors (drinking, smoking) and health status (rates of CVD, diabetes). Additionally, in the HABC study at baseline (1997–1998), older individuals with disability were excluded from participation in the study, rates of tooth loss were lower, prevalence of periodontal disease, and response rates were higher compared to the BRHS, which could also account for differences in associations. Worse health status, disability, and mortality (11% died during the follow‐up period), may have contributed to low response rates in the BRHS.^49^ As previously shown in the BRHS, participants who were healthier, from higher socioeconomic status, and those living close or with the ability to commute may have been more likely to participate in the 30‐year and subsequent examinations.^49^ Inclusion of healthier participants may have led to underestimation of reported associations. Furthermore, in older populations, disability status is changeable over time, and although we excluded participants with disability at baseline, it is possible that cases of incident disability may have experienced disability at some other point during the 2–4 years of follow‐up time. Moreover, because of the use of nonrecent data, rates of disability and oral health problems may differ from younger cohorts. It is also possible that edentulism (complete tooth loss) is associated with adaptation in ability to eat, and it is likely that the associations with disability might be different in those without natural teeth. Due to small numbers of edentulous participants, we were unable to examine this in our analysis. Even though we adjusted for several confounders, it is possible that due to measurement error, underreporting of confounders or unavailability of certain covariates, residual confounding may have been present.

In conclusion, this study reported limited associations between poor oral health and incident disability in older community‐dwelling adults. Findings were not consistent across the two studies; however, they provided some evidence that clinical and self‐reported oral health measures could be factors contributing to the onset of disability in older people. Nevertheless, more longitudinal studies are needed, where oral health and disability are assessed similarly. Additionally, the mechanistic pathways, which underpin this association merits investigation, to establish potential mediators which influence the association of poor oral health with onset of disability. Further understanding of these associations and mechanisms could identify ways that oral health could contribute to maintaining physical independence in older age.

## CONFLICT OF INTEREST

The authors declare no conflict of interest.

## AUTHOR CONTRIBUTIONS

Study concept and design: Eftychia Kotronia, Sheena E. Ramsay, Sasiwarang Goya Wannamethee, Olia Papacosta, and Peter H. Whincup. Acquisition of data: Sheena E. Ramsay, Sasiwarang Goya Wannamethee, Olia Papacosta, Peter H. Whincup, Lucy T. Lennon, and Robert J. Weyant. Analysis and interpretation of data: All authors. Drafting of the manuscript: All authors. Critical revision of the manuscript for important intellectual content: All authors.

## SPONSOR'S ROLE

The British Heart Foundation, Dunhill Medical Trust, and National Institutes of Health did not have a role in the design, methods, subject recruitment, data collections, analysis, and preparation of paper.

## Supporting information


**Table S1.** Details about covariates (type of variables/details of labeling)
**Table S2.** Odds ratios (OR) and 95% confidence interval (CI) for the associations of oral health problems with incident mobility limitations, activities of daily living (ADL), and instrumental activities of daily living (IADL) difficulties in older British men in the British Regional Health Study (BRHS).
**Table S3.** Odds ratios (OR) and 95% confidence interval (CI) for the association of oral health problems with incident mobility limitations and activities of daily living (ADL) difficulties in older US men and women in the Health, Aging and Body Composition (HABC) study.
