# Supplementary material for: Oral health problems and risk of incident disability in two studies of older adults in the United Kingdom and the United States
Source: J Am Geriatr Soc. 2022 Apr 19;70(7):2080–92. doi: 10.1111/jgs.17792 (PMC9283258; doi:10.1111/jgs.17792)
Supplement: Supplementary file 1 — Table S1. Details about covariates (type of variables/details of labeling) Table S2. Odds ratios (OR) and 95% confidence interval (CI) for the associations of oral health problems with incident mobility limitations, activities of daily living (ADL), and instrumental activities of daily living (IADL) difficulties in older British men in the British Regional Health Study (BRHS). Table S3. Odds ratios (OR) and 95% confidence interval (CI) for the association of oral health problems with incident mobility limitations and activities of daily living (ADL) difficulties in older US men and women in the Health, Aging and Body Composition (HABC) study. [file JGS-70-2080-s001.pdf]

Supplemental Text S1

Assessment of self-reported oral health problems

British Regional Heart Study

Self-rated oral health was assessed though the question "*Would you say that your* ***dental health*** *is*:". Available responses were excellent, good, fair, poor.

Health, Aging, and Body Composition Study

Self-rated oral health was assessed through the question "*How would you rate your overall oral health (teeth, gums, inside of mouth)?*". Available responses were excellent, good, fair, poor, don't know.

Limitation of food was assessed through the question: "*Do you limit the kinds or amounts of food you eat because of problems with your teeth or dentures?*". It indicates whether participants changed their eating habits due to dental issues. Chewing difficulties and difficulty eating were assessed through different questions.

Supplemental Text S2

Assessment of disability

Disability

Disability comprised mobility limitations, activities of daily living (ADL), and instrumental activities of daily living (IADL). In the BRHS, mobility limitations were defined as difficulty going up or down stairs or walking 400 yards. In particular, participants were asked: *Do you currently have difficulty carrying out any of the following activities on your own as a result of a long-term health problem? A) going up or down the stairs or B) Walking 400 yards*. Those who replied yes to either one of the two activities were classified as having mobility limitations, whereas those replying no did not have any mobility limitations. Moreover, to measure ADL difficulties, participants were asked whether they faced difficulties with any of the following activities: getting in and out of a chair, dressing and undressing yourself, bathing or showering, feeding yourself, including cutting food, or getting to and using the toilet. Available responses were no difficulty, some difficulty, need help or unable to do. Participants choosing some difficulty or unable to do/need help were classified as having ADL difficulties.[1] Similarly, for IADL problems, individuals were asked

whether they faced difficulties with any of the following activities :shopping for personal items, preparing your own meals, using telephone by yourself, managing money, or using public transport. Participants who replied some difficulty or unable to do/need help were classified as having IADL difficulties.[1] In the HABC Study, to assess mobility limitations participants were asked whether they experienced any difficulty walking 1 quarter of a mile or climbing 1 flight of stairs. Available responses were 0=unable to do, 1=very difficult, 2=somewhat difficult, 3=a little difficult, 4=not that easy, 5=somewhat easy, 6=very easy. Individuals choosing any reply between 0-3 for any of the two activities were classified as having mobility limitations. Additionally, to assess ADL problems, participants were asked: *Because of a health or physical problem do you have any difficulty doing the following activities*: dressing, getting in and out of bed, and/or bathing on your own. Available responses were yes, no or don't know. Those replying yes to having difficulty doing at least one of these activities were identified as having ADL difficulties.[1] Information on IADL was not collected in the HABC Study.

Supplemental Text S3

Assessment of depression

In the BRHS, during the 2010-2012 re-examination, depression was assessed through a questionnaire. The following question was used: *Have you ever been told by a doctor that you have or ever had depression?* Available responses were yes or no.

In the HABC Study, in Year 2 (1998-1999), prevalent depression was assessed through the use of anti-depressant medication (monoamine oxidase (MAO) inhibitors; non-topical tri/tetracyclic antidepressant; trazodone, bupropion, venlafaxine, nefaxodone-with reason referring to depression; selective serotonin reuptake inhibitors (SSRIs)-with reason referring to depression). Participants who were taking any of these medications were identified as having depression.

References

[1] Kotronia E, Wannamethee SG, Papacosta AO*, et al.* Oral Health, Disability and Physical Function: Results From Studies of Older People in the United Kingdom and United States of America. *Journal of the American Medical Directors Association*. 2019;**20**: 1654.e1651-1654.e1659.

Supplemental Table S1. Description of covariates in the BRHS and HABC Study.

Variables/Confounders	Type	Description/Categorization
		**BRHS**
Age	Continuous	Age at 30-year re-examination
Social class[a]	Binary	Manual; non-manual
Smoking	Categorical	Current smoker; ex-smoker; never smoker
Alcohol	Categorical	Daily; weekends only; occasionally once or twice/month; special occasions; none
Physical activity	Categorical	Inactive; occasional; light; moderate; moderate-vigorous; vigorous
History of CVD	Binary	Yes; no
History of diabetes	Binary	Yes; no
BMI	Categorical	Normal; overweight; obese
		**HABC Study**
Age	Continuous	Age at Year 2
Gender	Binary	Male; female
Race	Binary	White; African American
Education[a]	Categorical	Years of education. Less than high school; high school graduate; postsecondary
Smoking[b]	Categorical	Current smoker; ex-smoker; never smoker
Alcohol[b]	Categorical	More than 1 per day; 1-7 times per week; less than once per week; no consumption in past year
Physical Activity[b]	Continuous	Total kcal/kg/wk from household chores, walking and stairs, exercise or recreation activities and work or volunteering or caregiving
History of CVD	Binary	Yes; no
History of diabetes	Binary	Yes; no
BMI	Categorical	Normal; overweight; obese

[a] Measured at entry to the study (BRHS 1978-1980; HABC Study Year 1)
[b] Measured only at Year 1, not available at Year 2 of the HABC Study

Supplemental Table S2. Odds ratios (OR) and 95% CI for the associations of oral health problems with incident mobility limitations, ADL and IADL difficulties in older British men in the BRHS.

	Incident mobility limitations (n=175; 15%)		
	N (%)	Fully adjusted OR (95% CI)	Further adjusted for depression[b] OR (95% CI)
**Tooth Loss** (N of teeth)			
≥21	34 (9%)	1.00	1.00
15-20	35 (17%)	1.96 (1.12, 3.43)	2.07 (1.16, 3.72)
8-14	33 (22%)	2.02 (1.05, 3.91)	2.00 (1.05, 3.82)
1-7	13 (21%)	2.03 (0.94, 4.37)	1.86 (0.82, 4.24)
0	30 (18%)	1.52 (0.80, 2.92)	1.33 (0.69, 2.57)
**Periodontal disease (% of sites with loss of attachment >5.5 mm)**[c]			
<20%	71 (12%)	1.00	1.00
≥20%	38 (21%)	1.75 (1.04, 2.95)	1.61 (0.94, 2.77)
**Self-rated oral health**			
Good/Excellent	104 (14%)	1.00	1.00
Fair/Poor	67 (19%)	0.97 (0.64, 1.49)	1.01 (0.65, 1.57)
**Dry mouth symptoms**			
0	49 (11%)	1.00	1.00
1-2	62 (16%)	1.36 (0.84, 2.20)	1.33 (0.82, 2.18)
≥3	59 (22%)	2.08 (1.27, 3.42)	1.76 (1.03, 3.00)
**Cumulative oral health problems**[d]			
0	21 (9%)	1.00	1.00
1	78 (14%)	1.70 (0.99, 2.91)	1.40 (0.78, 2.49)
2	53 (21%)	1.43 (0.71, 2.87)	1.27 (0.65, 2.47)
≥3	23 (24%)	1.83 (0.70, 4.80)	2.27 (0.98, 5.29)
**Tooth Loss** (N of teeth)		**Incident ADL** (n=122; 11%)	
≥21	31 (7%)	1.00	1.00
15-20	29 (12%)	1.68 (.0.93, 3.03)	1.73 (.0.94, 3.16)
8-14	26 (16%)	1.75 (0.91, 3.36)	1.71 (0.86, 3.39)
1-7	10 (14%)	1.62 (0.70, 3.77)	1.83 (0.78, 4.33)
0	26 (14%)	1.60 (0.84, 3.01)	1.52 (0.78, 2.96)
**Periodontal disease (% of sites with loss of attachment >5.5 mm)**[c]			
<20%	63 (10%)	1.00	1.00
≥20%	29 (15%)	1.12 (0.64, 1.94)	1.09 (0.60, 1.97)
**Self-rated oral health**			
Good/Excellent	84 (10%)	1.00	1.00
Fair/Poor	63 (16%)	1.16 (0.76, 1.80)	1.21 (0.77, 1.90)
**Dry mouth symptoms**			
0	44 (9%)	1.00	1.00
1-2	48 (11%)	1.06 (0.64, 1.76)	1.04 (0.62, 1.76)
≥3	51 (16%)	1.73 (1.03, 2.90)	1.49 (0.86, 2.58)
**Cumulative oral health problems**[d]			
0	20 (8%)	1.00	1.00
1	59 (10%)	1.18 (0.64, 2.20)	1.23 (0.65, 2.33)
2	49 (17%)	2.10 (1.09, 4.04)	2.12 (1.07, 4.20)
≥3	22 (20%)	2.53 (1.14, 5.61)	2.52 (1.07, 5.96)
**Tooth Loss** (N of teeth)		**Incident IADL** (n=159; 12%)	
≥21	36 (8%)	1.00	1.00
15-20	28 (12%)	1.51 (0.83, 2.74)	1.68 (0.90, 3.13)

8-14	24 (14%)	1.48 (0.77, 2.84)	1.60 (0.81, 3.14)
1-7	8 (11%)	0.88 (0.33, 2.32)	0.98 (0.36, 2.70)
0	34 (18%)	1.56 (0.84, 2.91)	1.73 (0.90, 3.34)
**Periodontal disease (% of sites with loss of attachment >5.5 mm)[c]**			
<20%	62 (9%)	1.00	1.00
≥20%	27 (13%)	1.47 (0.85, 2.56)	1.56 (0.88, 2.76)
**Self-rated oral health**			
Good/Excellent	83 (10%)	1.00	1.00
Fair/Poor	72 (18%)	2.09 (1.34, 3.26)	1.91 (1.21, 3.03)
**Dry mouth symptoms**			
0	54 (11%)	1.00	1.00
1-2	57 (13%)	1.21 (0.73, 2.01)	1.08 (0.64, 1.84)
≥3	45 (14%)	1.33 (0.78, 2.27)	1.29 (0.74, 2.25)
**Cumulative oral health problems[d]**			
0	18 (7%)	1.00	1.00
1	87 (14%)	1.27 (0.68, 2.35)	1.34 (0.70, 2.54)
2	38 (13%)	1.30 (0.65, 2.60)	1.17 (0.56, 2.44)
≥3	18 (16%)	1.81 (0.78, 4.23)	2.15 (0.89, 5.19)

ADL: Activities of Daily Living; IADL: Instrumental Activities of Daily Living

[a] Adjusted for age, social class, smoking, alcohol, physical activity, history of CVD and diabetes, BMI

[b] Adjusted for age, social class, smoking, alcohol, physical activity, history of CVD and diabetes, BMI, depression; depression was assessed through a questionnaire. The following question was used: Have you ever been told by a doctor that you have or ever had depression? Available responses were yes or no.

[c] No associations were observed between pocket depth and incidence of disability; therefore only effect estimates for loss of attachment are presented

[d] <21 teeth, ≥3 dry mouth symptoms, difficulty eating, sensitivity to hot/cold/sweets

Supplemental Table S3. Odds ratios (OR) and 95% CI for the association of oral health problems with incident mobility limitations and ADL difficulties in older US men and women in the HABC Study.

	**Incident mobility limitations** (n=380; 20%)			**Incident ADL** (n=250; 13%)		
	N (%)	Fully adjusted OR (95% CI)	Further adjusted for depression[a] OR (95% CI)	N (%)	Age adjusted OR (95% CI)	Further adjusted for depression[b] OR (95% CI)
**Tooth Loss** (N of teeth)						
≥21	111 (16%)	1.00	1.00	84 (12%)	1.00	1.00
15-20	42 (17%)	0.86 (0.57, 1.31)	0.86 (0.57, 1.31)	31 (12%)	1.01 (0.65, 1.58)	0.80 (0.50, 1.27)
8-14	40 (23%)	1.05 (0.67, 1.66)	1.06 (0.67, 1.68)	23 (14%)	1.15 (0.70, 1.89)	0.89 (0.52, 1.52)
1-7	17 (15%)	0.56 (0.30, 1.02)	0.56 (0.31, 1.04)	11 (9%)	0.70 (0.36, 1.36)	0.58 (0.29, 1.15)
0	38 (31%)	1.86 (1.13, 3.06)	1.90 (1.15, 3.14)	17 (13%)	1.05 (0.60, 1.84)	0.77 (0.42, 1.44)
**Periodontal disease (% of sites with loss of attachment >3 mm)**[c]						
<20%	49 (16%)	1.00	1.00	31 (10%)	1.00	1.00
≥20%	73 (15%)	0.85 (0.56, 1.31)	0.85 (0.55, 1.30)	54 (10%)	0.99 (0.62, 1.58)	0.90 (0.54, 1.48)
**Self-rated oral health**						
Good/Excellent	238 (18%)	1.00	1.00	146 (11%)	1.00	1.00
Fair/Poor	106 (22%)	1.09 (0.83, 1.45)	1.10 (0.83, 1.45)	81 (16%)	1.59 (1.19, 2.14)	1.43 (1.05, 1.96)
**Dry mouth**						
No	331 (19%)	1.00	1.00	218 (12%)	1.00	1.00
Yes	13 (27%)	1.45 (0.73, 2.89)	1.49 (0.74, 2.98)	9 (16%)	1.41 (0.68, 2.92)	1.34 (0.63, 2.83)
**Cumulative oral health problems**[d]						
0	87 (15%)	1.00	1.00	66 (12%)	1.00	1.00
1	174 (19%)	0.98 (0.72, 1.33)	0.98 (0.72, 1.33)	109 (12%)	1.02 (0.74, 1.42)	0.82 (0.58, 1.17)
2	62 (25%)	1.35 (0.91, 2.02)	1.35 (0.91, 2.02)	36 (15%)	1.33 (0.86, 2.07)	1.08 (0.68, 1.71)
≥3	27 (28%)	1.50 (0.87, 2.57)	1.51 (0.88, 2.58)	21 (18%)	1.68 (0.98, 2.87)	1.28 (0.72, 2.28)

ADL: Activities of Daily Living

[a] Adjusted for age, gender, race, education, smoking, alcohol, physical activity, history of CVD and diabetes, BMI

[b] Adjusted for age, gender, race, education, smoking, alcohol, physical activity, history of CVD and diabetes, BMI, depression; depression was assessed through the use of anti-depressant medication (monoamine oxidase (MAO) inhibitors; non-topical tri/tetracyclic antidepressant; trazodone, bupropion, venlafaxine, nefaxodone-with reason referring to depression; selective serotonin reuptake inhibitors (SSRIs)-with reason referring to depression). Participants who were taking any of these medications were identified as having depression.

[c] No associations were observed between pocket depth and incidence of disability; therefore only effect estimates for loss of attachment are presented

[d] <21 teeth, dry mouth when eating, difficulty eating or chewing, limitation of food due to gum problems
